# Polygonatum Polysaccharide Regulates Macrophage Polarization and Improves LPS-Induced Acute Lung Injury through TLR4-MAPK/NF-*κ*B Pathway

**DOI:** 10.1155/2022/2686992

**Published:** 2022-07-14

**Authors:** Weizheng Zhou, Jiang Hong, Tao Liu, Mengxing Li, Hai Jin, Xiaowei Wang

**Affiliations:** Department of Thoracic Surgery, Changhai Hospital, Shanghai 200433, China

## Abstract

**Objective:**

To investigate the effects of polygonatum sibiricum polysaccharides (PSPs) on the polarization of macrophages to M1 and M2 phenotypes and their potential mechanism.

**Methods:**

PSPs samples were prepared through water extraction and alcohol precipitation assay. The properties of PSPs were identified and analyzed by high-performance liquid chromatography, FT-IR, and NMR assay. Then, the effects of PSPs on mouse macrophage RAW264.7 viability were measured by CCK-8 assay. The cells were randomly divided into the control group, PSPs group, LPS group, and LPS + PSPs group. M1 phenotype polarization of RAW264.7 cells was induced by LPS treatment. The effects of various treatments on expression of M2 phenotype CD206, activation of TLR4-MAPK/NF-*κ*B signal pathway, and translocation of NF-*κ*B into the nucleus were determined by ELISA, western blot, and immunofluorescence assay, respectively. TLR4 inhibitor, TAK-242, and MAPK inhibitor, BIRB 796, were used to verify the effects of PSPs on the TLR4-MAPK/NF-*κ*B pathway. The mice model of acute lung injury (ALI) was established and randomly divided into control group, PSPs group, LPS group, and LPS + PSPs group. Bronchoalveolar lavage fluid (BALF) and lung tissue were collected to measure protein, inflammatory cells, neutrophil and macrophage cells number, and the levels of IL-6 and TNF-*α* in BALF. Flow cytometry and western blot assay measured the phenotypic changes of macrophages and the activation of the TLR4-MAPK/NF-*κ*B signaling pathway.

**Results:**

The concentrations of PSPs lower than 100 *μ*g/mL showed no toxicity to RAW264.7 cells. PSPs treatment could significantly reverse the reduction of CD206 protein expression (*P* < 0.05) and the increase of the expression of inflammatory factor TNF-*α*, IL-1*β*, and IL-6 (all *P* < 0.05), TLR4-MAPK/NF-*κ*B signaling pathway activation (all *P* < 0.05), and NF-*κ*B translocation into the nucleus induced by LPS. The effect of inhibitors TAK-242 and BIRB 796 was consistent with that of PSPs. In the mice model of ALI, PSPs treatment could reduce the total protein levels of BALF and the number of inflammatory cells level, reverse the number changes of neutrophils and macrophages, and downregulate the proinflammatory factors IL-6 and TNF-*α* caused by LPS (all *P* < 0.05). In addition, PSPs treatment could also significantly reverse the increase in the number of iNOS expressing macrophages in alveolar lavage fluid induced by LPS (*P* < 0.05). In contrast, CD206-expressed cells decreased (*P* < 0.05). PSPs could also reverse LPS-induced TLR4-MAPK/NF-*κ*B signal pathway protein activation (all *P* < 0.05).

**Conclusion:**

PSPs could suppress TLR4-MAPK/NF-*κ*B activation induced by LPS, inhibit M1 phenotypic polarization of macrophages, and promote M2 phenotypic polarization, thus playing an anti-inflammatory role.

## 1. Introduction

The inflammatory response caused by infection or tissue damage is the first line of defense against pathogens from invading human body [1]. Although the inflammatory response acts as a defensive system in vivo, excessive inflammatory response can lead to severe tissue damage [2]. Sepsis, caused by overactivated inflammatory response, is one of the most common causes of death in intensive care units [3]. Inflammation, immune dysregulation, and coagulation dysfunction are known to be involved in the development of sepsis; other than that, persistent activation of macrophages contributes significantly in acceleration sepsis development [4].

Macrophages exhibit significant heterogeneity. As an indispensable part of innate immunity, they play a key role in inflammatory response. Activated macrophages can be differentiated into two phenotypes, the classical activation (M1) phenotype and the alternating activation (M2) phenotype [5]. The activation of the M1 phenotype elevates the level of secretions induced by oxidative stress, thus promoting inflammation and leading to tissue damage [6]. In contrast, activation of the M2 phenotype increases the production of anti-inflammatory factors, thereby reducing inflammation and promoting tissue repair [7]. Most importantly, macrophages play an essential role in the development of sepsis. M1 macrophages secrete multiple inflammatory mediators to stimulate cytokine secretion and septic shock, which could lead to tissue damage and even death in severe situation [8]. When macrophages polarize into the M2 phenotype, it contributes significantly to onset symptoms and development control of sepsis [9]. It is known that regulating macrophage polarization can affect the inflammatory response of sepsis. However, few compounds, as a treatment method, could promote the polarization of macrophages to M2 phenotype [10]. Further, the underlying mechanism of macrophage polarization is complicated and remains uninvestigated. Liu et al. found that M1 macrophage polarization depended on TLR4-MAPK/NF-*κ*B signaling pathway. The inhibition of the TLR4-MAPK/NF-*κ*B signaling pathway can suppress the polarization of M1 macrophages and further induce M2 type macrophages polarization [11]. Yang et al. reported that curcumin nanoparticles which inhibit TLR4-MAPK/NF-*κ*B signaling pathway could inhibit M1 macrophage polarization [12]. Taken both studies together, TLR4-MAPK/NF-*κ*B signaling pathway played a critical role in macrophage polarization.

Polygonatum sibiricum polysaccharides (PSPs) are mainly composed of four monosaccharides with different chemical properties. They were initially extracted from the dried rhizomes of the flowering plant polygonatum sibiricum. Recent pharmacological studies have shown that PSPs have a variety of biological activities, including antioxidant, antiaging, antifatigue, immune enhancement, antibacterial, and anti-inflammatory activities [13]. Previous studies have shown that PSPs can inhibit TLR4-MAPK/NF-*κ*B signaling pathway with an antitumor role [14]. TLR4 signaling pathway has been found to be closely related to the occurrence and development of acute lung injury (ALI) [15]. In addition, TLR4 is known to be expressed in both immune cells (such as macrophages and neutrophils) and nonimmune cells (such as alveolar endothelial cells and epithelial cells). Previous studies have shown that TLR4-mediated inflammatory response played a key role in lipopolysaccharide (LPS)-induced ALI [16]. However, whether PSPs could affect macrophage polarization and its underlying mechanism remain uninvestigated.

In this study, we aimed to first explore the effect of PSPs on polarization of macrophages to M1 and M2 phenotypes. With the determined effects, further studies were conducted to evaluate its potential underlying mechanism. In addition, an ALI mice model induced by LPS was established to observe the anti-inflammatory and other protective effects of PSPs in ALI.

## 2. Materials and Methods

### 2.1. Extraction, Preparation, and Identification of PSPs

PSPs were extracted, prepared, ad identified based on the method of [17]. In brief, PSPs samples were prepared by water extraction and alcohol precipitation using ultrapure acetone, Sevag reagent, and activated carbon. After extraction, 200 mg of sample was dissolved in 2 ml distilled water, and DEAE Sepharose FastFlow was performed at a 2 ml/min flow rate. Four components were obtained, named PSP1, PSP2, PSP3, and PSP4 in turn. The four components' homogeneity and average molecular weight were determined by high-performance molecular exclusion chromatography, and the monosaccharide components were analyzed by high-performance liquid chromatography precolumn derivatization. Fourier transform infrared spectrophotometer was used for infrared spectral analysis (FT-IR). Nuclear magnetic resonance spectroscopy (NMR) was applied to qualitatively analyze the composition and structure of various organic and inorganic substances. PSPs mixtures were used in formal experiments.

### 2.2. Cell Culture

Mouse RAW264.7 macrophages were purchased from Shanghai Fuheng Biotechnology Co., Ltd. Macrophages were cultured with DMEM medium (Corning, USA) containing 10% fetal bovine serum (GIBCO, USA) and 1% penicillin-streptomycin (GIBCO, USA) in a constant temperature incubator at 37°C and 5% CO_2_ relative humidity. The cells were subcultured to prepare for subsequent experiments when reaching 80–90% confluency.

### 2.3. Cell Activity Assay

RAW264.7 cells were seeded into 96 well plates (Corning, USA) at the density of 10^4^ cells/well and treated with different concentrations of PSPs (0 (control group), 5, 10, 25, 50, 100, 200, and 400 *μ*g/mL) for 24 h. After 24 h, 10 *μ*L CCK-8 reagent (Dojindo, Japan) was added, and cells were incubated for 2 hours prior to being placed into the spectrophotometer (Bio-Rad, USA) to measure the absorbance value (OD) at 450 nm. The relative cell viability was calculated using the following formula: relative cell viability = (average OD value of treatment group−background OD value)/(average OD value of control group−background OD value).

### 2.4. Western Blot for Protein Expression

RIPA lysing buffer (Beyotime, China) was used to lyse RAW264.7 cells and mice lung tissue. After splitting on ice for 30 min, samples were centrifuged at 4°C under 10000 g for 20 min. The protein concentration was then determined using BCA Kit (Beyotime, China) according to the manufacturer's guidance. The protein was isolated from 10–12% SDS-PAGE gel electrophoresis and then transferred to the PVDF membrane (Millipore, USA). The membrane was blocked with blocking solution (Beyotime, China) at room temperature for 1 h and incubated with diluted primary antibody at 4°C overnight. The primary antibody included TLR4 (#14358, 1 : 2000, Cell Signaling Technology, USA), MAPK (p38, #9212, 1 : 2000, Cell Signaling Technology, USA), phosphorylated MAPK (p-p38, #4511, 1 : 1000, cell signaling technology, USA) and phosphorylated NF-*κ*B (p-NF-*κ*B, #3033, 1 : 1000, Cell Signaling Technology, USA), NF-*κ*B (#8242, 1 : 2000, Cell Signaling Technology, USA), CD206 (#24595, 1 : 1000, Cell Signaling Technology, USA), and *β*-actin (#3700, Cell Signaling Technology, USA). The membrane was washed three times with Tris buffer (TBS, Beyotime, China) containing 0.1% Tween-20 (Beyotime, China) on the next day. The membrane was then incubated with the corresponding secondary antibody on a shaking agitator at room temperature for one hour. The fluorescence intensity of protein bands was observed in the imaging system with an enhanced chemiluminescence kit (ECL, Millipore, USA). Then, the grayscale of protein bands was analyzed quantitatively with ImageJ software.

### 2.5. Enzyme-Linked Immunosorbent Assay (ELISA)

RAW264 cells were collected according to instructions. The concentrations of TNF-*α*, IL-1*β*, IL-6, and IL-10 were determined by ELISA kit (Nanjing Jiancheng Bioengineering Institute, China).

### 2.6. Immunofluorescence Assay

RAW264.7 cells were seeded at a density of 2 × 10^4^ cells/well for corresponding treatment. The cells were fixed with 4% paraformaldehyde (Beyotime, China) at room temperature for 15 minutes, infiltrated with 0.5% Triton X-100 (Solarbio, China) for 10 minutes, rinsed with precooled PBS for 3 times, and then blocked with 5% goat serum (Beyotime, China) for 1 hour. Cells were incubated with NF-*κ*B primary antibody (#8242, 1 : 500, Cell Signaling Technology, USA) overnight at 4°C. The fixed cells were washed three times with TBST the following day, and the corresponding IgG secondary antibody combined with Alexa fluor 594 was applied for 1 h at room temperature in the dark. The nuclei were stained with DAPI (Beyotime, China), and the images were captured by a fluorescence microscope (Olympus, Japan).

### 2.7. Establishment of the ALI Animal Model

Male C57BL/6 mice (6–8 weeks old, 25–30 g) were purchased from Changzhou Cavens Experimental Animal Co., Ltd. (Changzhou, China). Mice were hosted in an environment with a light/dark cycle of 12/12 hours and appropriate temperature (23 ± 2°C), drinking and eating freely. The ethics committee has approved all operations related to animal feeding and treatment in this study of our hospital. The mice were randomly divided into four groups (10 mice in each group). Normal saline and PSPs (200 mg/kg/day) [14] were administered by gavage every day. After three consecutive days, 10 mg/kg LPS [18] or normal saline was injected intraperitoneally. After 6 hours, the mice were anesthetized with ketamine/xylazine (150 : 10 mg/kg) mixture, bronchoalveolar lavage was performed, bronchoalveolar lavage fluid (BALF) was collected, and the whole lung was retained for follow-up study. To count BALF cells, cells were concentrated using Cytospin 4 (ThermoFisher Scientific, USA) and stained using the Shandon Kwik Diff kit (ThermoFisher Scientific, USA) and counted under a light microscope (Olypus, Japan).

### 2.8. Flow Cytometry

BALF was treated with cell supernatant (ThermoFisher Scientific, USA) to obtain alveolar macrophages, and the cells were resuspended with staining buffer (BD Biosciences, USA). FCR blocker (BD Biosciences, USA) was added to the cells for incubation to increase the specificity of the antibody. Firstly, the cells were stained with the marker (F4/80, mature mouse macrophage surface specific marker antibody), then fixed and infiltrated with fixed osmotic buffer (Beyotime, China), and then stained with anti-CD206 (#24595, 1 : 5000, Cell Signaling Technology, USA) or anti-iNOS antibody (#13120, 1 : 5000, Cell Signaling Technology, USA). Then, F4/80 CD206 or F4/80 iNOS double-stained macrophages were detected by flow cytometer, the horizontal axis was the F4/80 fluorescence channel, and the vertical axis was the CD206 or iNOS fluorescence channel, which was analyzed using FlowJo software.

### 2.9. Statistical Analysis

All measurement data in this study were expressed as mean ± standard deviation. SPSS 18.8 (SPSS, USA) was used for statistical analysis. Student's *t*-test was used for the comparison between the two groups. Under the same condition, a one-way analysis of variance (ANOVA) was used to compare different groups. *P* < 0.05 was considered statistically significant.

## 3. Results

### 3.1. Extraction, Identification, and Analysis of PSPs

**Figure 1:**
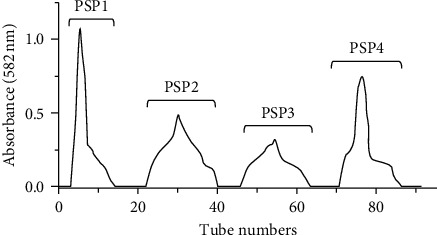
Ion exchange chromatography of PSP components. PSP1 was washed with deionized water, and PSP2, PSP3, and PSP4 were eluted with different concentrations of NaCl (0.05, 0.2, and 0.5 M).

PSPs were obtained by water extraction and alcohol precipitation. After deproteinization and decolorization, PSP1, PSP2, PSP3, and psp4 were obtained through anion exchange chromatography, as shown in Figure 1. The average molecular weights of the four components were around 4.436, 2.278, 7.783, and 6.562 kDa, respectively. The four components consisted of Galactose, Rhamnose, Mannose, Glucose, and Xylose but in different proportions. The details of different compositions are shown in Table 1.

**Figure 2:**
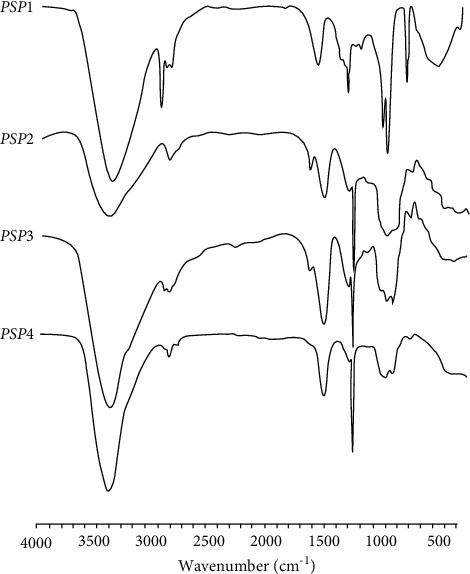
FI-IR analysis of PSPs components.

As shown in Figure 2, the absorption peaks of PSP1, PSP2, PSP3, and PSP4 in the FT-IR spectrum range of 4000 to 500 cm^−1^ were as follows. (1) A characteristic strong absorption peak caused by the tensile vibration of O−H bond could be seen around 3400 cm^−1^. (2) Weak absorption peaks caused by stretching and bending vibration of C−H bonds (C−H, CH_2_, CH_3_) could be seen at about 2900 cm^−1^ and 1300 cm^−1^. (3) At about 1700 cm^−1^ and 1650 cm^−1^, the stretching bands of C=O group and carboxylic acid bond (COO^−^) were seen, respectively. (4) There was a weak absorption peak between 1200 and 1000 cm^−1^, corresponding to C−O glycosidic bond, indicating the existence of pyran ring. (5) The characteristic absorption peak at 850 cm^−1^ might be caused by the vibration of isomer C−H group. Analyzation of absorption peaks suggested that PSP1, PSP2, PSP3, and PSP4 were different in molecular vibration characteristics and molecular structures.

### 3.2. The Effect of PSPs on RAW264.7 Cell Activity

**Figure 3:**
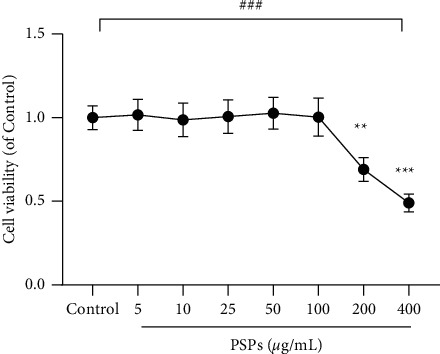
Effect of PSPs on RAW264.7 cell activity. Compared with control group, ^*∗∗*^*P* < 0.01, ^*∗∗∗*^*P* < 0.001; comparison within different groups, ^###^*P* < 0.001.

As shown in Figure 3, different concentrations of PSPs (control group of 0 *μ*g/mL, 5, 10, 25, 50, 100, 200, and 400 *μ*g/mL) were used to treat RAW264.7 cells for 24 hours. The cell activity was then measured by the CCK-8 method. There was no effect on cell activity observed with PSPs concentration lower than 100 *μ*g/mL. When PSP concentration reached 200–400 *μ*g/mL, RAW264.7 cell activity decreased significantly, suggesting that high concentrations (higher than 100 *μ*g/mL PSPs) exhibited potential cellular toxicity. Therefore, 100 *μ*g/mL was used as the treatment concentration of PSPs in the follow-up study otherwise indicated.

### 3.3. The Effect of PSPs on RAW264.7 Cells on the Secretion of Inflammatory Factors

**Figure 4:**
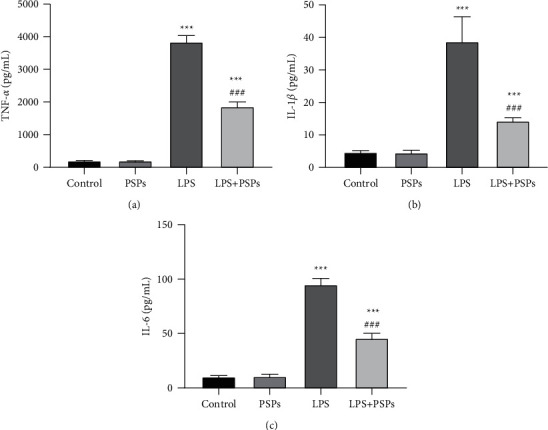
The effect of PSPs on RAW264.7 cells on the secretion of inflammatory factors. ^*∗∗∗*^*P* < 0.001 compared with control group; ^###^*P* < 0.001, compared with LPS group.

As shown in Figure 4, RAW264.7 was treated with PSPs and LPS. After 24 hours, the results indicated that TNF-*α*, IL-1*β*, and IL-6 in the LPS and LPS + PSPs groups were significantly higher than those in the control group (all *P* < 0.05). Compared with the LPS only group, cotreatment with PSPs could reduce TNF- *α*, IL-1*β*, and IL-6 levels (all *P* < 0.05), suggesting that PSPs treatment could reverse the LPS-mediated secretion of TNF-*α*, IL-1*β*, and IL-6 in RAW264.7 cells.

### 3.4. The Effect of PSPs on Macrophage M2 Phenotypic Polarization in RAW264.7 Cells

**Figure 5:**
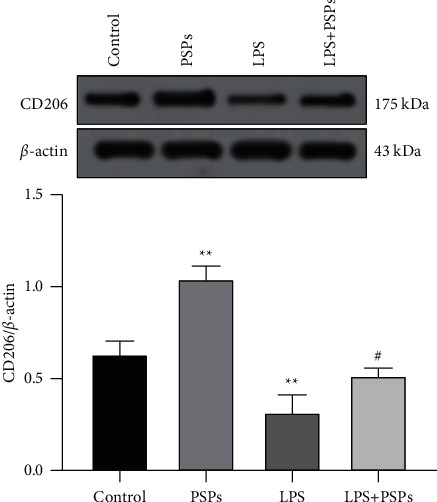
Effect of PSPs on M2 phenotypic polarization of RAW264.7 cells. Compared with control group, ^*∗∗*^*P* < 0.01; compared with LPS group, ^#^*P* < 0.05.

As shown in Figure 5, PSPs-treated RAW264.7 increased the expression level of M2 phenotypic marker CD206 compared with control (*P* < 0.05). In comparison, the expression of CD206 protein was inhibited by LPS (*P* < 0.05). In addition, PSPs cotreatment could significantly increase the expression level of CD206 compared to the LPS group (*P* < 0.05), suggesting that PSPs could promote M2 phenotypic polarization of macrophages.

### 3.5. The Effect of PSPs on the TLR4-MAPK/NF-*κ*B Signaling Pathway

**Figure 6:**
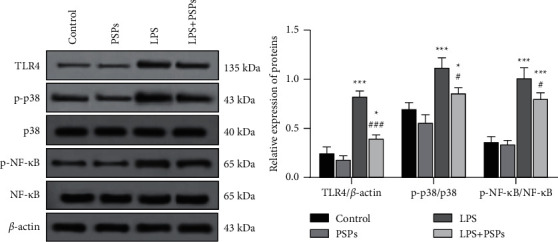
Effect of PSPs on the TLR4-MAPK/NF-*κ*B signaling pathway. Compared with control group, ^*∗*^*P* < 0.05, ^*∗∗∗*^*P* < 0.001; compared with LPS group, ^#^*P* < 0.05, ^###^*P* < 0.001.

LPS significantly increased the protein expression of TLR4, phosphorylated MAPK (p-p38), and phosphorylated NF-*κ*B (p-NF-*κ*B) (all *P* < 0.05) as shown in Figure 6, indicating induction of TLR4-MAPK/NF-*κ*B signaling pathway by LPS. When treated with PSPs simultaneously, the induced activation of LPS was inhibited (*P* < 0.05). Compared with the LPS group, the expression level of TLR4, phosphorylated p38, and phosphorylated NF-*κ*B in LPS + PSPs group decreased (all *P* < 0.05), suggesting that PSPs contributed to the inhibition of TLR4-MAPK/NF-*κ*B signaling pathway activation.

**Figure 7:**
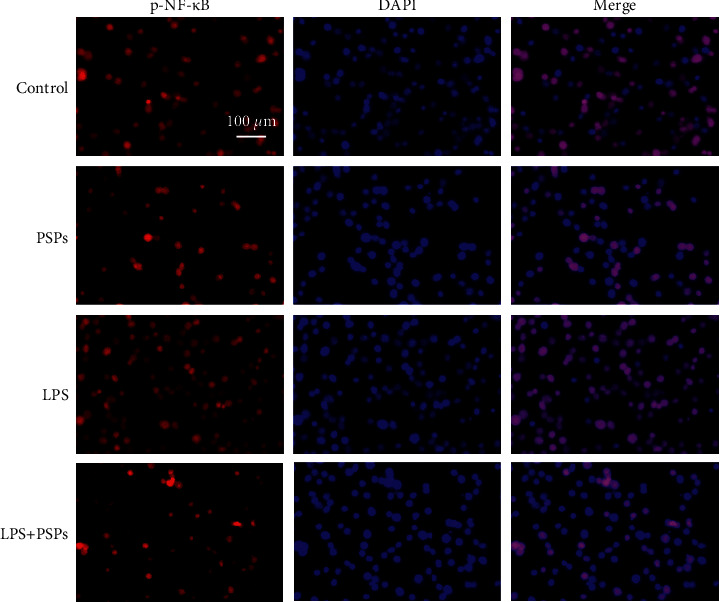
Effect of PSPs treatment on NF-*κ*B nuclear translocation. Magnification: 10x, scale: 100 *μ*m.

As shown in the fluorescent images in Figure 7, the red fluorescence marks the NF-*κ*B positive cells that transported into the nucleus, and the blue fluorescence shows all living cells. It can be seen that PSPs treatment reduced the translocation of NF-*κ*B into the nucleus (*P* < 0.05), while LPS-induced NF-*κ*B transport into the nucleus (*P* < 0.05). With cotreatment of LPS and PSP, the induction of LPS could be significantly reversed (*P* < 0.05).

### 3.6. TLR4 Inhibitor, TAK-242, and MAPK Inhibitor, BIRB 796, Were Used to Verify the Inhibitory Effect of PSPs on the MAPK/NF-*κ*B Signaling Pathway

**Figure 8:**
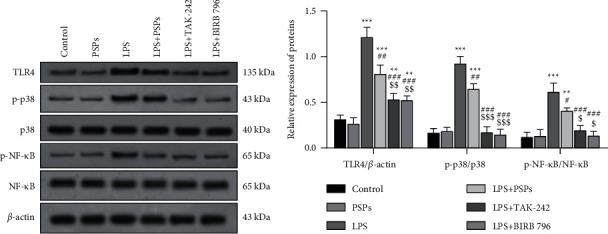
Verification of PSPs impact on TLR4-MAPK/NF-*κ*B signaling pathway. Compared with control group, ^*∗∗*^*P* < 0.01, ^*∗∗∗*^*P* < 0.001; compared with LPS group, ^#^*P* < 0.05, ^##^*P* < 0.01, and ^###^*P* < 0.001; compared with LPS + PSPs group, ^$^*P* < 0.05, ^$$^*P* < 0.01, and ^$$$^*P* < 0.001.

As shown in Figure 8, LPS significantly increased the protein expression of TLR4 and the phosphorylation of p38 and NF-*κ*B (*P* < 0.05). Compared with the LPS group, PSPs treatment could significantly reverse the negative effects (all *P* < 0.05). In addition, the administration of pathway inhibitors TAK-242 and BIRB 796 could also inhibit TLR4-MAPK/NF-*κ*B signaling pathway (all *P* < 0.05). In addition, compared with the LPS + PSPs group, the inhibitory effects of TAK-242 and BIRB 796 on signal pathway activation were more pronounced (all *P* < 0.05).

**Figure 9:**
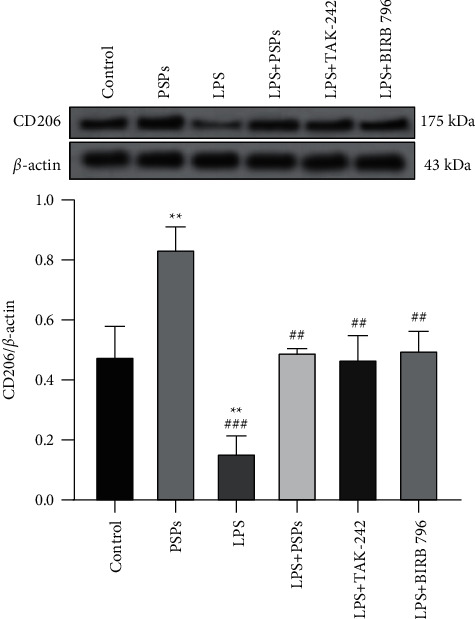
Verification of PSPs impact on M2 cell phenotype in RAW264.7 cells. Compared with control group, ^*∗∗*^*P* < 0.01; compared with LPS group, ^##^*P* < 0.01, ^###^*P* < 0.001.

In addition, as shown in Figure 9, PSPs treatment could significantly increase the expression of CD206 protein (*P* < 0.05), while LPS had the opposite effect (*P* < 0.05). The inhibitory effect of LPS on the expression of CD206 protein was reversed by inhibitors TAK-242 and BIRB 796 (*P* < 0.05), and the inhibitory effect was similar to that of PSPs (all *P* < 0.05) in cells cotreated with LPS and PSPs.

### 3.7. Effects of PSPs on Inflammatory Response in ALI Mice

**Figure 10:**
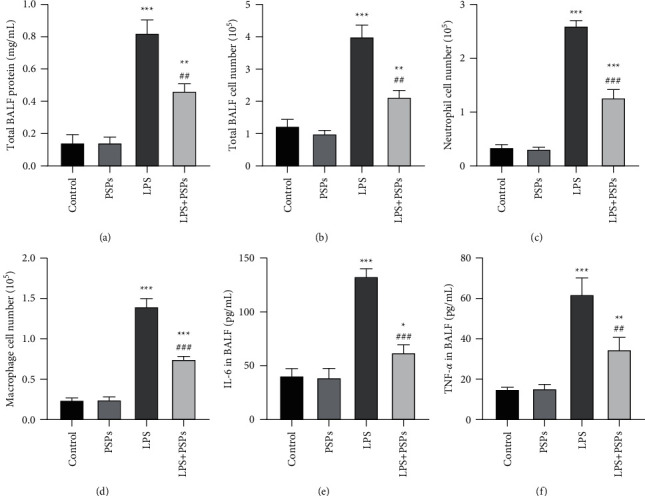
BALF analysis of the effect of PSPs on the inflammatory response in ALI mice. (a) The total protein concentration in BALF of each group. (b) The total number of inflammatory cells in BALF in each group. (c) The number of neutrophils in BALF in each group. (d) The number of macrophages in BALF in each group. (e) IL-6 levels in BALF of each group. (f) TNF-*α* levels in BALF of each group. Compared with the control group, ^*∗*^*P* < 0.05, ^*∗∗*^*P* < 0.01, and ^*∗∗∗*^*P* < 0.001; compared with the LPS group, ^#^*P* < 0.05, ^##^*P* < 0.01, and ^###^*P* < 0.001.

In this study, a mice model was used to further evaluate the therapeutic effect of PSPs on LPS-induced ALI in vivo. As shown in Figures 10(a) and 10(b), by measuring the protein level and the total number of inflammatory cells in BALF, we found that, compared with the LPS group, the protein level of the LPS + PSPs group decreased (*P* < 0.05), and the total number of inflammatory cells decreased (*P* < 0.05), suggesting that the inflammatory response was alleviated. In addition, this study also determined the number of neutrophils and macrophages and the levels of proinflammatory cytokines IL-6 and TNF-*α* in the BALF of mice in each group. As shown in Figures 10(c)–10(f), treatment with PSPs reversed the changes in the number of neutrophils and macrophages caused by LPS and also decreased IL-6 and TNF-*α* levels in BALF compared with the LPS group. All the above results suggested that PSPs have a therapeutic effect on LPS-induced ALI. Off note, the evaluated indicators also showed successful establishment of LPS-induced ALI mice model.

### 3.8. Effects of PSPs on M1 and M2 Phenotypic Polarization and TLR4-MAPK/NF-*κ*B Signaling Pathway

**Figure 11:**
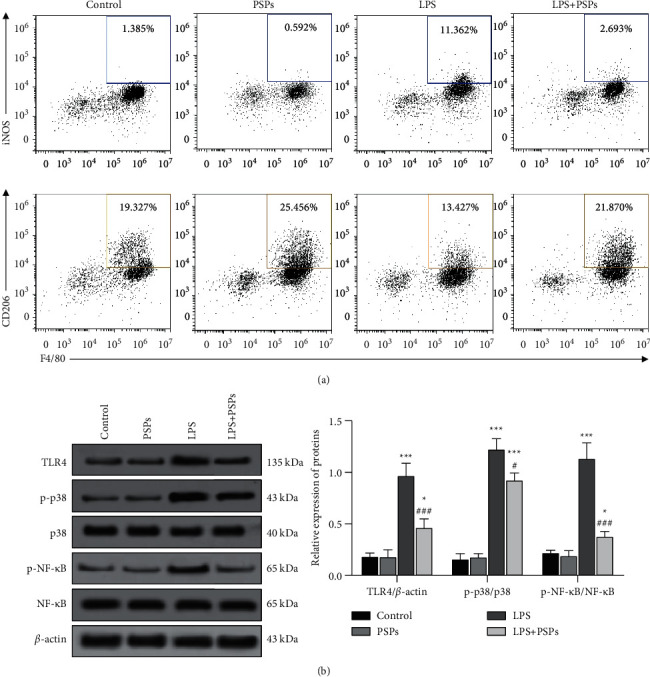
Effects of PSPs on M1 and M2 phenotypic polarization and TLR4-MAPK/NF-*κ*B signaling pathway of mouse macrophages. (a) The effect of PSPs on M1 and M2 phenotypic polarization of mouse macrophages was measured by flow cytometry. (b) The expression of TLR4-MAPK/NF-*κ*B signal proteins in lung tissue. Compared with the control group, ^*∗∗*^*P* < 0.01; compared with LPS group, ^##^*P* < 0.01, ^###^*P* < 0.001.

It is known that F4/80 was a classic mature mouse macrophage surface specific marker used to target mouse macrophages, while iNOS and CD206 are markers of M1 and M2 macrophages, respectively. As shown in Figure 11(a), the number of F4/80^+^ iNOS^+^ expressing macrophages in the PSPs treatment group decreased (*P* < 0.05), while the number of F4/80^+^ CD206^+^ macrophages increased (*P* < 0.05). LPSs resulted in an increase in F4/80^+^ iNOS^+^ expressing cells (*P* < 0.05), while the number of F4/80^+^ CD206^+^ macrophages decreased (*P* < 0.05). Compared with the LPS group, the number of F4/80^+^ iNOS^+^ expressing cells could be significantly reduced by cotreatment with PSPs (*P* < 0.05); however, the number of F4/80^+^ CD206^+^ cells increased under the same condition (*P* < 0.05).

In addition, as shown in Figure 11(b), LPS could significantly induce TLR4, p-p38, and p-NF-*κ*B in mice lung tissue compared to control (all *P* < 0.05). Meanwhile, PSP treatment could dramatically reverse the above changes (all *P* < 0.05).

## 4. Discussion

Research evidence illustrated that macrophage-mediated inflammation played a crucial role in inflammatory diseases such as acute lung injury and sepsis [19, 20]. Macrophages are characterized by high diversity and plasticity and can be divided into M1 and M2 phenotypes. M2-polarized macrophages are anti-inflammatory and protective, while M1-polarized macrophages are believed to have adverse effects on cells [21]. Previous research identified that compounds isolated from polygonatum had anti-inflammatory effects [22, 23]. In the past, Liu et al. found that PSPs could downregulate the LPS-mediated increase in neutrophil ratio and reduce the level of LPS-induced inflammatory factors in BALF of the rat ALI model [22]. In addition, Liu et al. reported that PSPs could inhibit the TLR4/NF-*κ*B pathway, thereby reducing LPS-induced apoptosis of normal human bronchial epithelial cells [22]. However, according to our knowledge, no study investigated the effect of PSPs on macrophage polarization and whether macrophages can mediate the protective and anti-inflammatory effect of PSPs in ALI.

This study found that PSPs could significantly polarize macrophages and derive the M2 anti-inflammatory phenotype. M1 or M2 polarization of macrophages can be characterized by the expression of M1- or M2-related markers [24]. CD206 is known to be a unique phenotypic marker of M2 macrophages, and iNOS and TNF-*α* are the markers of M1 macrophages. To study the effects of LPS and PSPs on M1 and M2 phenotypic polarization of macrophages, RAW264.7 was stimulated by LPS. In addition, cotreatment of PSPs and LPS was administrated. It was found that PSPs could significantly promote the expression of CD206 and inhibit the increase of TNF-*α*, IL-1*β*, and IL-6 levels caused by LPS, suggesting that PSPs could reverse the M1 polarization of macrophages stimulated by LPS. Further, PSPs could reduce the inflammatory factors secretion by macrophages induced by LPS. In addition, this study also established a mice ALI model. The expression of CD206 and iNOS in macrophages in BALF was measured by flow cytometry. It was also found that PSPs could reduce the number of macrophages expressing iNOS and increase the number of cells expressing CD206. In mice model, consistent with cells study, PSPs could promote M2 polarization of macrophages and play an anti-inflammatory role.

TLR is a transmembrane transduction receptor that transmits LPS signals from extracellular to intracellular space. In addition, TLR4 can directly bind to LPS and activate NF-*κ*B and MAPK signaling pathways, which cause the synthesis and release of various inflammatory mediators and ultimately trigger and amplify the inflammatory response [25]. As one of the primary inflammatory regulators, NF-*κ*B is a crucial transcription factor that promotes the transcription of genes encoding proinflammatory cytokines. Drugs or inhibitors can exert protective and immunomodulatory functions by inhibiting NF-*κ*B signaling [26]. Studies have found that NF-*κ*B signaling pathway inhibition could promote the polarization of M2 macrophages and stimulate the activation of NF-*κ*B showing contrary effects [27]. In addition, MAPK is known to be a threonine/serine kinase that played a key role in inflammation by regulating NF-*κ*B-dependent gene transcription [28]. Previous studies have found that glucosamine might play an anti-inflammatory role by inhibiting the phosphorylation of MAPK protein which hinders the M1 polarization of local macrophages [29]. Therefore, this study examined the TLR4-MAPK/NF-*κ*B signaling pathway expression in macrophage polarization. As found in previous studies, LPS could activate the TLR4-MAPK/NF-*κ*B signaling pathway. Further cotreatment with PSPs could significantly inhibit the expression of TLR4 protein, phosphorylated MAPK, and NF- *κ*B protein. Meanwhile, PSPs treatment could also inhibit the translocation of NF-*κ*B protein into the nucleus. Furthermore, when TLR4 inhibitor and MAPK inhibitor were given simultaneously, the inhibitory effect on this pathway was similar to that of PSPs, and the M2 polarization of CD206 protein increased. Taken together, PSPs might act as a natural inhibitor of TLR4-MAPK/NF-*κ*B pathway activity to play an anti-inflammatory role.

Although this study explored the effect of PSPs on LPS-induced macrophage polarization in ALI and its underlying mechanism, there were certain limitations. M2-type macrophages are known to have anti-inflammatory effects, while M1-type macrophages are known to have proinflammatory effects. Therefore, the inflammation in ALI aggravates the transformation of M1-type macrophages to M2-type, which play a certain role in reducing lung injury. It is possible to develop chronic pulmonary fibrosis when ALI does not have a timely and effective intervention, resulting in the continued existence of lung tissue damage. It is worth noting that M2 macrophages have profibrotic effects such as promoting the deposition of extracellular matrix components [30]. However, fibrosis in lung tissue is not only associated with macrophages. Other factors such as airway and alveolar epithelial cells and fibroblasts can also produce cytotoxic/proinflammatory and profibrotic mediators [31]. It can be seen that chronic pulmonary fibrosis is a complex process. Based on this study, although it is known that PSPs have anti-inflammatory and lung-protecting effects such as reducing ALI, the effect of PSPs on chronic pulmonary fibrosis and their mechanism still need to be further explored.

## 5. Conclusion

PSPs hindered the M1 phenotypic polarization of macrophages and promoted the differentiation of M2 phenotypic polarization by inhibiting the LPS-dependent TLR4-MAPK/NF-*κ*B signaling pathway, which plays an essential anti-inflammatory role. However, it is still unclear about the protective role of PSPs in the lung. Therefore, further population research may be needed to demonstrate the anti-inflammatory and lung-protective role of PSPs as the natural inhibitor of the TLR4-MAPK/NF-*κ*B signaling pathway. PSPs could inhibit the LPS-mediated activation of the TLR4-MAPK/NF-*κ*B signaling pathway, which inhibited the M1 phenotypic polarization of macrophages and promoted the differentiation of M2 phenotypic polarization. However, whether PSPs acting as the natural inhibitor of TLR4-MAPK/NF-*κ*B signaling pathway could contribute to an anti-inflammatory and lung-protective role needs to be demonstrated by further population research.

## Figures and Tables

**Table 1:** Molecular weight and chemical composition of PSPs.

Component	PSP1	PSP2	PSP3	PSP4
Molecular weight (kDa)	4.436	2.278	7.783	6.562
Glycosyl composition (mol%)				
Mannose	15.84	—	1.22	1.81
Rhamnose	—	20.57	58.55	73.16
Glucose	2.34	1.94	1.65	-
Galactose	81.82	75.36	36.97	19.33
Xylose	—	2.13	1.61	5.70

## Data Availability

The data used to support the findings of this study are included within the article.
